# The Psychological Impact of Diabetes as a Risk Factor for Erectile Dysfunction at the Layla Qasim Center in Erbil City, Iraq

**DOI:** 10.7759/cureus.73415

**Published:** 2024-11-10

**Authors:** Abdulqader Hussein Hamad

**Affiliations:** 1 Department of Psychiatric and Mental Health Nursing, College of Nursing, Hawler Medical University, Erbil, IRQ

**Keywords:** diabetes mellitus, erectile dysfunction, mental health associate, psychological impact, risk factors

## Abstract

Background

Diabetes mellitus is a chronic metabolic disorder that can lead to various complications, including erectile dysfunction (ED). Therefore, this study aimed to investigate the relationship between ED and psychological factors (anxiety, stress, and depression) in Erbil City, Iraq.

Methodology

Using a purposive sampling method, this cross-sectional study was conducted from June 15th to November 27th, 2023, at the Layla Qasim Center in Erbil City. The questionnaire included demographic information, the Depression, Anxiety and Stress Scale - 21 Items for assessing depression, anxiety, and stress, and the International Index of Erectile Dysfunction Questionnaire. Statistical analysis was performed using Stata version 12 (StataCorp LLC, College Station, TX, USA). Significance levels were considered at p-values <0.05.

Results

A total of 403 participants were included in the study. The mean score for depression was 9.95 ± 4.99, indicating moderate levels of depression. Anxiety levels were more severe, with a mean score of 9.25 ± 4.25, while stress levels were moderate with a mean score of 11.63 ± 3.91. ED scores indicated mild-to-moderate ED, with a mean score of 13.46 ± 4.30. There was a significant negative correlation between ED and depression (r = -0.11, p < 0.001), anxiety (r = -0.16, p < 0.001), and stress (r = -0.13, p < 0.001).

Conclusions

The study demonstrated a significant negative correlation between ED, depression, anxiety, and stress among diabetic patients. Policymakers and healthcare providers should develop targeted interventions to address psychological factors and support ED in diabetic patients.

## Introduction

Diabetes mellitus is a chronic metabolic disorder characterized by elevated blood glucose levels, which can lead to various complications affecting multiple organ systems [1]. One of the lesser-discussed but significant complications of diabetes is erectile dysfunction (ED), defined as the persistent inability to achieve or maintain an erection sufficient for satisfactory sexual performance [2]. The relationship between diabetes and ED has been well-established, with studies consistently showing a higher prevalence of ED among men with diabetes compared to the general population [3,4]. This increased prevalence can be attributed to the multifactorial etiology of ED in diabetes involving a complex interplay of vascular, neurological, and endocrine factors [4]. However, the psychological impact of diabetes on ED is often overlooked, despite evidence suggesting that psychological factors play a crucial role in its development and maintenance. Additionally, comorbidities such as obesity, dyslipidemia, and hypertension, often secondary to diabetes, increase the risk and severity of ED, compounded by lifestyle factors such as poor diet and lack of exercise. Furthermore, side effects from diabetes medications and iatrogenic causes due to inexperienced management highlight the need for a holistic approach to treating diabetic patients with ED [5,6]. Diabetes can have a profound effect on an individual’s mental health, with increased rates of depression, anxiety, and stress reported among people with diabetes [6,7]. These psychological factors, in turn, can contribute to the development of ED and exacerbate the condition’s impact on quality of life [8,9].

Moreover, the psychological burden of ED itself can be substantial, leading to reduced self-esteem, relationship difficulties, and further deterioration of physical and mental health [10-13]. The interplay between diabetes, ED, and psychological factors creates a vicious cycle that can significantly impact an individual’s overall well-being. Despite the recognized importance of addressing psychological aspects in the management of ED, there is a lack of research specifically examining the psychological impact of diabetes as a risk factor for ED in the context of Middle Eastern populations. This is particularly relevant as the Middle East, particularly Iraq, has witnessed a significant increase in the prevalence of diabetes in recent years [14]. For instance, in Erbil City, the capital of the Kurdistan Region of Iraq, diabetes has become a major public health concern, with a reported prevalence of 13.9% among adults [15-17]. This high prevalence of diabetes in the region underscores the importance of addressing its complications, including ED. Recent findings also point to broader psychosocial issues in Erbil’s population, with studies indicating high levels of internet addiction and poor sleep quality among secondary school students, conditions associated with psychosocial challenges such as stress and anxiety [18]. Addressing these psychological aspects, however, requires culturally adapted therapeutic interventions.

Cognitive behavioral therapy (CBT), in particular, is a versatile therapeutic approach that has shown effectiveness across a range of mental health conditions, including suicide prevention, anxiety, and depression [19]. CBT can also be beneficial for patients experiencing ED, particularly when psychological factors such as anxiety and stress contribute to the condition [20]. By addressing maladaptive thought patterns and behaviors, CBT can help patients manage the emotional and cognitive aspects of ED, reducing psychological distress and improving sexual health outcomes. Moreover, CBT techniques can be adapted to accommodate cultural sensitivities, making it a valuable intervention in contexts where discussing sexual health may be challenging [21]. Given its effectiveness, CBT presents a promising approach for supporting men with ED in Erbil City, especially those affected by the psychological burden of diabetes.

Cultural factors may also play a significant role in shaping attitudes, beliefs, and behaviors related to sexual health and mental well-being [22-24]. In many Middle Eastern societies, including Iraq, discussions about sexual health issues such as ED are often considered taboo, leading to a reluctance to seek help and a lack of awareness about available treatments [11,25,26]. Additionally, traditional gender roles and societal expectations can contribute to the psychological burden experienced by men with ED [27]. These cultural factors highlight the need for culturally sensitive approaches to understanding and addressing the psychological impact of diabetes and ED in this population. Furthermore, the ongoing political instability, economic challenges, and social disturbance in Iraq have had a profound impact on the population’s mental health. Exposure to conflict, displacement, and loss can exacerbate psychological distress, which may contribute to the development and maintenance of ED among men with diabetes [28]. Therefore, the unique sociopolitical context of Erbil City and its potential influence on the psychological well-being of individuals with diabetes and ED warrants further investigation.

Despite the growing recognition of the psychological impact of diabetes and ED, there is a significant gap in the literature concerning this relationship in the context of Erbil City and the broader Middle Eastern region. The majority of research on the psychological dimensions of diabetes and ED has been undertaken in Western nations, with no attention given to the distinctive cultural, social, and political elements that may affect this correlation in Middle Eastern cultures. Furthermore, existing studies often rely on small sample sizes or do not utilize validated psychological assessment tools, which limits the generalizability and reliability of their findings. The lack of comprehensive, culturally relevant research on the psychological impact of diabetes as a risk factor for ED in Erbil City underscores the necessity for further investigation in this area. Therefore, this study aims to assess the psychological status of diabetes and evaluate ED in this context. Additionally, I seek to examine the relationship between ED and each component of psychological status, specifically anxiety, stress, and depression.

## Materials and methods

Study design, setting, and period

This cross-sectional study was conducted at the Layla Qasim Center in Erbil City. The purposive sampling method was used to collect data from June 15, 2023, to November 27, 2023.

Sample size

The sample size was calculated with a 95% confidence interval, an estimated response distribution of 50%, and a 5% margin of error. Using the formula for an infinite population, a Zα/2 value of 1.96 was determined. Although the initial sample size calculation was 385 participants, the sample was expanded to 403 participants due to the availability of additional cases.

Inclusion and exclusion criteria

The study’s inclusion criteria were individuals diagnosed with diabetes mellitus who were currently undergoing treatment at the Layla Qasim Center in Erbil City and were willing to participate. Exclusion criteria included patients experiencing recurrent complications of diabetes, such as diabetic neuropathy, nephropathy, or retinopathy, which could independently affect the quality of life and confound the study’s focus on psychological impacts specific to ED. Additionally, individuals with documented psychological disorders, including major depressive disorder, generalized anxiety disorder, or any other diagnosed psychiatric condition, were excluded to maintain focus on psychological factors associated primarily with diabetes and ED. Refusal to participate was also grounds for exclusion.

Study tools and data collection

The questionnaire was divided into three main parts. The first part gathered demographic data, including age, date of marriage, diabetes mellitus period, body mass index (BMI), education, occupation, area of residence, economic status, and marital relationship. The second part consisted of the Depression, Anxiety and Stress Scale - 21 Items (DASS-21), which is the short version of the Depression, Anxiety, and Stress Scale (DASS) and consists of 21 items designed to measure levels of depression, anxiety, and stress in individuals. This tool is commonly used in clinical and research settings to assess emotional state and help identify psychological problems. The third part was the International Index of Erectile Dysfunction (IIEF-5) Questionnaire, which contained five items assessing ED. To ensure accuracy, the questions were translated from English to Kurdish using a forward-backward method, and the translation was verified by a psychiatrist. Data were collected by distributing questionnaires to participants who met the inclusion criteria, with each participant being allotted a total of 15-20 minutes to complete the questionnaire.

Pilot study

The study questionnaires were tested in an initial study with 30 participants from the general population between April 5th and May 5th, 2023, to assess the internal consistency and reliability of the items before they were used in the actual study. The internal consistency of the items was calculated using Cronbach’s alpha [29]. For the DASS-21, the overall Cronbach’s alpha was 0.91, indicating excellent internal consistency. For the IIEF-5, the overall Cronbach’s alpha was 0.88, demonstrating very good reliability. Additionally, to ensure the validity of the content of the tools, they were reviewed by 10 nursing professors. The data from this initial study were excluded from the final analysis.

Measures

Sociodemographic Characteristics

Various sociodemographic variables were measured during the study. These included age, date of marriage, diabetes mellitus period, BMI, education, occupation, area of residence, economic status, and marital relationship.

The DASS-21 Questionnaire

The second part of the questionnaire was the DASS-21 [30], designed to assess emotional states related to depression, anxiety, and stress. This instrument comprises the following three subscales: Depression, Anxiety, and Stress, each containing seven items tailored to assess specific emotional states. The Depression scale targets symptoms such as dysphoria and hopelessness, the Anxiety scale focuses on autonomic arousal and situational anxiety, while the Stress scale evaluates chronic arousal indicators such as difficulty relaxing and nervous arousal. Participants rated each item on a four-point Likert scale. Scores for depression, anxiety, and stress were obtained by summing up the scores for the relevant items in each subscale. The DASS-21 adopts a dimensional approach to quantify symptom severity, with scoring ranges for depression (normal: 0-4, mild: 5-6, moderate: 7-10, severe: 11-13, extremely severe: 14+), anxiety (normal: 0-3, mild: 4-5, moderate: 6-7, severe: 8-9, extremely severe: 10+), and stress (normal: 0-7, mild: 8-9, moderate: 10-12, severe: 13-16, extremely severe: 17+). The internal consistency of the DASS-21 was assessed using Cronbach’s alpha [29], which showed an excellent reliability score of 0.91.

The IIEF-5 Questionnaire

The third part of the questionnaire was the IIEF-5 Questionnaire [31], designed to assess ED. This instrument comprises five items that evaluate different aspects of ED, namely, confidence in getting and keeping an erection, frequency of erections hard enough for penetration, ability to maintain an erection after penetration, difficulty maintaining an erection to completion of intercourse, and satisfaction with sexual intercourse. Participants rated each item on a scale from 1 to 5, with higher scores indicating better ED. The IIEF-5 score is the sum of the ordinal responses to the five items, with scores ranging as follows: 22-25 indicating no ED, 17-21 indicating mild ED, 12-16 indicating mild-to-moderate ED, 8-11 indicating moderate ED, and 5-7 indicating severe ED. The internal consistency of the IIEF-5 was assessed using Cronbach’s alpha [29], which showed a very good reliability score of 0.88.

Ethical approval and informed consent

This study followed the Institutional Research Ethics Board and the Declaration of Helsinki guidelines. The Ethical approval was obtained from the College of Nursing at Hawler Medical University on May 14, 2023, with the code number 30. Written informed consent was obtained from all participants before they filled out the questionnaires.

Statistical analysis

Data were summarized and reported with frequency and percentage for qualitative variables. Quantitative variables with a normal distribution were presented with mean and standard deviations. The data were weighted to the population and standardized according to WHO population estimates for 2000-2025 using survey analysis. Given that the data were normally distributed, I have maintained the logical flow of the analysis and have adhered to parametric tests such as Pearson’s correlation, followed by multiple linear regression. The relationship between ED with depression, anxiety, and stress was assessed using Pearson’s correlation coefficient. The adjusted association between these variables and other confounding factors was evaluated using multiple linear regression analysis. Data analysis was performed using Stata version 12 (StataCorp LLC, College Station, TX, USA), with significance levels considered at p-values <0.05.

## Results

Demographic and clinical characteristics

A total of 403 participants were included in the study. The ages of the participants ranged from 31 to 80 years, with a mean age of 55.59 ± 9.88 years. The distribution of the date of marriage varied, with the majority (150, 37.2%) married between the ages of 26 and 30 years, and the mean date of marriage was 31.19 ± 5.15 years. Regarding the duration of diabetes mellitus, the mean period was 10.68 ± 6.37 years, with 127 (31.5%) participants having diabetes for 6-10 years. The BMI of participants indicated that nearly half (192, 47.6%) were in the overweight category (25-29.9 kg/m^2^), with a mean BMI of 26.31 ± 3.63 kg/m^2^. Educational levels showed a diverse distribution, with 104 (25.8%) being illiterate and 109 (27.1%) having primary education. Occupational status revealed that 195 (48.4%) were self-employed, while 72 (17.9%) were unemployed. The majority of the participants resided in urban areas (361, 89.6%). Regarding economic status, 205 (50.9%) participants reported their status as insufficient. Marital relationships were generally positive, with 291 (72.2%) rating them as good. Depression levels among participants indicated a mean score of 9.95 ± 4.99, classifying as moderate. Anxiety levels were more severe, with a mean score of 9.25 ± 4.25. Stress levels were moderate, with a mean score of 11.63 ± 3.91. ED scores indicated mild-to-moderate ED, with a mean score of 13.46 ± 4.30 (Table 1).

**Table 1:** Demographic and clinical profile of study participants. F = Frequency; % = percentage; SD = standard deviation

Variables	Characteristics (n = 403)	F	%
Age (years)	31–40	25	6.2
	41–50	102	25.3
	51–60	140	34.7
	61–70	116	28.8
	71–80	20	5.0
	Mean ± SD	55.59 ± 9.88	
Date of marriage	21–25	48	11.9
	26–30	150	37.2
	31–35	117	29.0
	36–40	80	19.9
	41–50	8	2.0
	Mean ± SD	31.19 ± 5.15	
Diabetes mellitus period (years)	1–5	120	29.7
	6–10	127	31.5
	11–15	72	17.9
	16–20	60	14.9
	21–25	24	6.0
Body mass index (kg/m^2^)	˂18.5	17	4.3
	18.5–24.5	94	23.3
	25–29.9	192	47.6
	˃30	100	24.8
	Mean ± SD	26.31 ± 3.63	
Education	Illiterate	104	25.8
	Read and write	48	11.9
	Primary	109	27.1
	secondary	69	17.1
	Academic	37	9.2
	Others	36	8.9
Occupation	Employed	56	13.9
	Self-employed	195	48.4
	Retired	80	19.9
	Unemployed	72	17.9
Area of residence	Urban	361	89.6
	Rural	42	10.4
Economic status	Sufficient	60	14.9
	Somehow sufficient	138	34.2
	Insufficient	205	50.9
Marital relationship	Bad	16	4.0
	Good	291	72.2
	Very good	96	23.8
Depression	Normal	28	6.9
	Mild	52	12.9
	Moderate	152	37.7
	Severe	113	28.0
	Extremely severe	58	14.4
	Mean ± SD	9.95 ± 4.99	
Anxiety	Normal	36	8.9
	Mild	56	13.9
	Moderate	52	12.9
	Severe	72	17.9
	Extremely severe	187	46.4
	Mean ± SD	9.25 ± 4.25	
Stress	Normal	64	15.9
	Mild	76	18.9
	Moderate	92	22.8
	Severe	109	27.0
	Extremely severe	62	15.4
	Mean ± SD	11.63 ± 3.91	
Erectile dysfunction	No erectile dysfunction	17	4.2
	Mild erectile dysfunction	105	26.1
	Mild-to-moderate erectile dysfunction	145	36.0
	Moderate erectile dysfunction	104	25.8
	Severe erectile dysfunction	32	7.9
	Mean ± SD	13.46 ± 4.30	

Correlation between ED and psychological variables

Pearson’s correlation analysis revealed significant negative correlations between ED and psychological variables. Specifically, ED showed a weak negative correlation with depression (r = -0.11, p < 0.001), anxiety (r = -0.16, p < 0.001), and stress (r = -0.13, p < 0.001). These results suggest that lower ED is associated with higher levels of depression, anxiety, and stress (Table 2).

**Table 2:** Correlation between erectile dysfunction and psychological variables. Significance was set at p < 0.05.

Variable pair	Pearson correlation (r)	Sample size (n)	Significance (p-value)
Erectile function – depression	-0.11	403	<0.001
Erectile function – anxiety	-0.16	403	<0.001
Erectile function – stress	-0.13	403	<0.001

Correlation between ED, psychological, and demographic variables

The multiple linear regression analysis examined the association between ED and various demographic and psychological variables. Age (B = -0.92, p < 0.001) and date of marriage (B = -1.01, p < 0.001) were significantly negatively associated with ED, indicating that older age and later dates of marriage are associated with lower ED. Similarly, the duration of diabetes mellitus (B = -0.40, p = 0.09) showed a negative association, though not statistically significant. BMI (B = 0.73, p < 0.001) was positively associated with ED, suggesting that a higher BMI is related to better ED. Educational level (B = 0.09, p = 0.46) and occupation (B = -0.14, p = 0.53) did not show significant associations. Area of residence (B = -1.84, p < 0.001) and marital relationship (B = -2.00, p < 0.001) were significantly negatively associated with ED, indicating that living in urban areas and having poorer marital relationships are linked to lower ED. Economic status (B = -0.23, p = 0.41) did not show a significant association. Among the psychological variables, depression (B = -0.06, p < 0.001), anxiety (B = -0.08, p < 0.001), and stress (B = -0.06, p < 0.001) were all significantly negatively associated with ED, suggesting that higher levels of depression, anxiety, and stress are associated with lower ED (Table 3).

**Table 3:** Final model of multiple linear regression for assessing the association between erectile dysfunction, depression, anxiety, stress, and demographic variables. Note: Erectile function is the dependent variable, Significance was set at p < 0.05.

Variables	Coefficient standardized (B)	Coefficient unstandardized (B)	95% confidence interval		P-value
			Lower	Upper	
Age	-0.21	-0.92	-1.38	-0.47	<0.001
Date of marriage	-0.23	-1.01	-1.46	-0.55	<0.001
Diabetes mellitus period	-0.08	-0.40	-0.64	0.05	0.09
Body mass index	0.14	0.73	0.21	1.26	<0.001
Education	0.04	0.09	-0.16	0.34	0.46
Occupation	-0.03	-0.14	-0.56	0.29	0.53
Area of residence	-0.13	-1.84	-3.10	-0.57	<0.001
Economic status	-0.04	-0.23	-0.78	0.32	0.41
Marital relationship	-0.23	-2.00	-2.88	-1.13	<0.001
Depression	-0.13	-0.06	-0.17	-0.03	<0.001
Anxiety	-0.19	-0.08	-0.21	-0.05	<0.001
Stress	-0.14	-0.06	-0.17	-0.03	<0.001

## Discussion

This study aimed to investigate the relationship between ED and psychological factors such as depression, anxiety, and stress in Erbil City, Iraq. Overall, the results revealed a significant negative correlation between ED, depression, anxiety, and stress among diabetic patients, highlighting the complex interplay between these factors and their impact on sexual health. These findings emphasize the multifaceted challenges faced by individuals with diabetes, particularly regarding their sexual and mental health.

Diabetes mellitus is a chronic condition that affects millions of people worldwide, and its prevalence continues to rise [32]. The condition is associated with various complications, including ED, which can significantly impact the quality of life of affected individuals [33]. In particular, the psychological aspects of this relationship have not been explored, especially in the context of Iraq. Therefore, given the importance of understanding the multifaceted nature of ED in diabetic patients, this study aimed to shed light on the associations between ED and psychological factors such as depression, anxiety, and stress in Erbil City.

The demographic characteristics of the study participants provide valuable insights into the population affected by diabetes and ED in Erbil. Specifically, the wide age range, with an average in the mid-50s, aligns with global trends indicating a higher prevalence of diabetes and its complications in older individuals [34]. Furthermore, the predominance of married participants diagnosed with diabetes for a significant duration highlights the long-term impact of the condition on sexual health and relationships. Additionally, the diverse educational backgrounds and high proportion of overweight individuals in our study are consistent with previous research linking lower education levels and obesity to an increased risk of diabetes and its complications [35].

The psychological profile of our participants, characterized by moderate levels of depression and stress and more severe levels of anxiety, underscores the substantial mental health burden associated with diabetes and ED. These insights are crucial as these findings are in line with previous studies that have reported high rates of psychological distress among individuals with chronic conditions, particularly those affecting sexual dysfunction [36,37]. Thus, the similarity in psychological profiles across different populations highlights the universal impact of these conditions on mental well-being. However, the specific cultural and societal factors in Iraq may contribute to the unique manifestations and coping mechanisms observed in our study population.

A key finding of our study is the significant negative correlation between ED and psychological variables such as depression, anxiety, and stress. This critical relationship indicates that as ED declines, the levels of these psychological issues tend to increase. In fact, these results are consistent with previous research that has demonstrated the bidirectional nature of the association between sexual dysfunction and mental health [38]. Consequently, the consistency of this relationship across different studies and populations underscores the importance of addressing both physical and psychological aspects of sexual health in the management of diabetes and its complications.

The multiple linear regression analysis further highlighted the complex interplay between demographic factors, ED, and psychological well-being. Specifically, the influence of variables such as age, marital status, education level, and economic status on the relationship between ED and mental health aligns with previous research [39]. These observations underline the need for a holistic approach to the assessment and management of ED in diabetic patients, taking into account the individual’s sociodemographic background and its potential impact on psychological well-being. Therefore, the strong association between ED and psychological factors, coupled with the influence of various demographic variables, has important implications for clinical practice and public health interventions. In particular, our findings suggest that comprehensive care for diabetic patients should incorporate both medical management of ED and psychological support to address depression, anxiety, and stress. Moreover, the integration of mental health services into diabetes care pathways could help improve sexual dysfunction and overall quality of life for affected individuals. In addition, public health initiatives aimed at promoting healthy lifestyles and preventing diabetes should also address the psychological aspects of the condition to reduce the burden of ED and its associated mental health consequences.

While this study provides valuable insights into the relationship between ED and psychological factors in diabetic patients, it is not without limitations. This study was conducted in a single center in Erbil City, which may limit the generalizability of the findings to other regions of Iraq or different cultural contexts. Future research should, therefore, aim to replicate these findings in larger, more diverse samples and employ longitudinal designs to better understand the temporal dynamics of the associations between ED and psychological well-being in diabetic patients. Moreover, exploring the effectiveness of interventions that simultaneously address both physical and mental health aspects of ED could provide valuable insights for clinical practice and policy development.

## Conclusions

The study demonstrated a significant negative correlation between ED, depression, anxiety, and stress among diabetic patients. It is recommended for policymakers and healthcare providers to develop targeted interventions to address psychological factors and support erectile function in diabetic patients. Such interventions could include routine psychological screenings and the incorporation of mental health support into diabetes management plans. Additionally, increasing awareness and education about the psychological impact of diabetes and ED can help reduce stigma and encourage patients to seek timely help.
